# Destined for destruction: The role of methionine aminopeptidases and plant cysteine oxidases in N-degron formation

**DOI:** 10.1093/plphys/kiae667

**Published:** 2025-01-28

**Authors:** Andrea Fuentes-Terrón, Rebecca Latter, Samuel Madden, Isabel Manrique-Gil, Jessenia Estrada, Noelia Arteaga, Inmaculada Sánchez-Vicente, Oscar Lorenzo, Emily Flashman

**Affiliations:** Department of Botany and Plant Physiology, Facultad de Biología, Instituto de Investigación en Agrobiotecnología (CIALE), Universidad de Salamanca, C/Río Duero 12, Salamanca 37185, Spain; Department of Chemistry, University of Oxford, 12 Mansfield Road, Oxford OX1 3TA, UK; Department of Earth Sciences, University of Oxford, South Parks Road, Oxford OX1 3AN, UK; Department of Biology, University of Oxford, South Parks Road, Oxford OX1 3RB, UK; Department of Botany and Plant Physiology, Facultad de Biología, Instituto de Investigación en Agrobiotecnología (CIALE), Universidad de Salamanca, C/Río Duero 12, Salamanca 37185, Spain; Department of Botany and Plant Physiology, Facultad de Biología, Instituto de Investigación en Agrobiotecnología (CIALE), Universidad de Salamanca, C/Río Duero 12, Salamanca 37185, Spain; Department of Botany and Plant Physiology, Facultad de Biología, Instituto de Investigación en Agrobiotecnología (CIALE), Universidad de Salamanca, C/Río Duero 12, Salamanca 37185, Spain; Department of Botany and Plant Physiology, Facultad de Biología, Instituto de Investigación en Agrobiotecnología (CIALE), Universidad de Salamanca, C/Río Duero 12, Salamanca 37185, Spain; Department of Botany and Plant Physiology, Facultad de Biología, Instituto de Investigación en Agrobiotecnología (CIALE), Universidad de Salamanca, C/Río Duero 12, Salamanca 37185, Spain; Department of Biology, University of Oxford, South Parks Road, Oxford OX1 3RB, UK

## Abstract

The cysteine/arginine (Cys/Arg) branch of the N-degron pathway controls the stability of certain proteins with methionine (Met)-Cys N-termini, initiated by Met cleavage and Cys oxidation. In seeding plants, target proteins include the Group VII Ethylene Response Factors, which initiate adaptive responses to low oxygen (hypoxic) stress, as well as Vernalization 2 (VRN2) and Little Zipper 2 (ZPR2), which are involved in responses to endogenous developmental hypoxia. It is essential that these target proteins are only degraded by the N-degron pathway under the appropriate physiological conditions. Modification of their N-termini is under enzymatic control by Met Aminopeptidases (MetAPs) and Plant Cysteine Oxidases (PCOs); therefore, the substrate-binding requirements and catalytic effectiveness of these enzymes are important for defining which Met-Cys–initiating proteins are degraded. Physiological conditions can also impact the activity of these enzymes, and the well-characterized oxygen sensitivity of the PCOs ensures target proteins are stabilized in hypoxia. In this review we compile the functional and structural properties of MetAPs and PCOs, including their interactions with substrates. We also consider the evolution of MetAPs and PCOs through the plant kingdom to highlight their important role in controlling the initial steps of this branch of the N-degron pathway.

## Introduction

**Figure 1.**
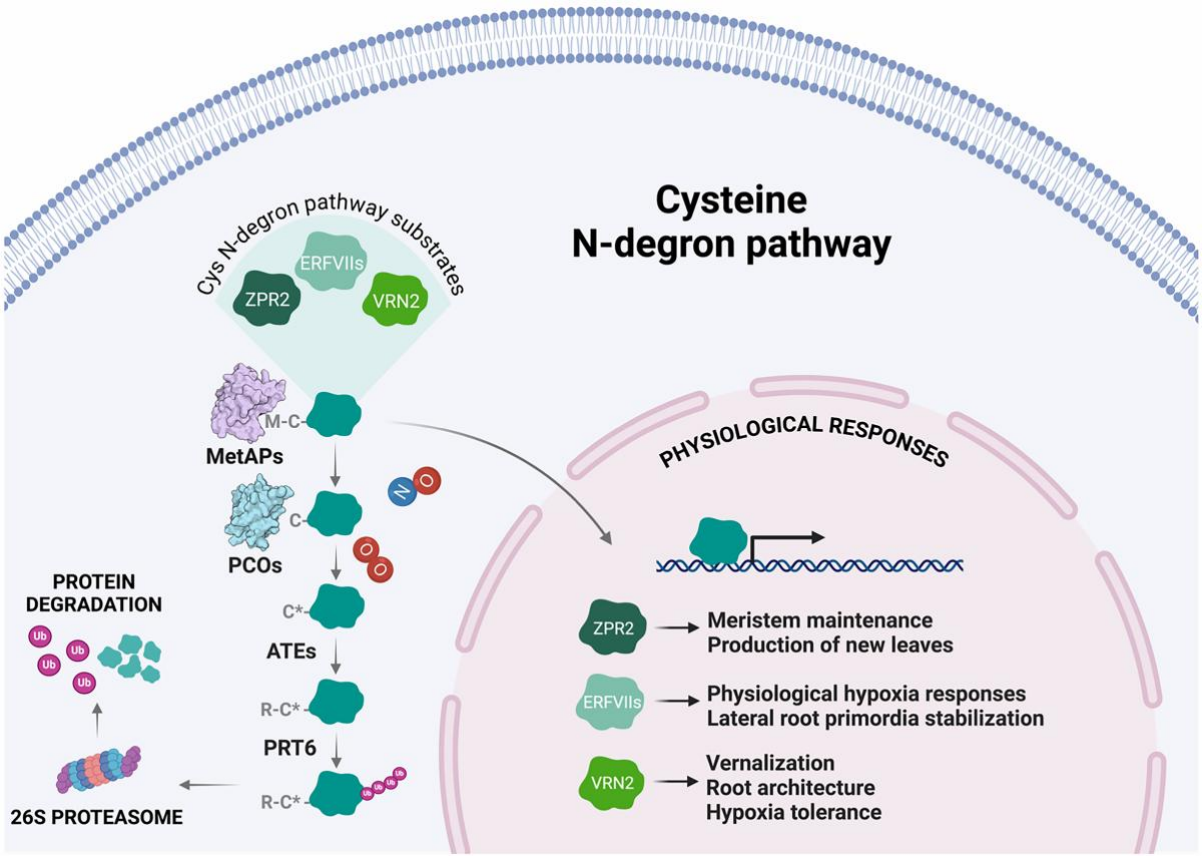
Physiological effects related to MetAPs and PCOs substrates characterized in plants. ZPR2 (Weits et al. 2019), ERFVIIs (Weits et al. 2014), and VRN2 (Gibbs et al. 2018) stability is controlled by the Cys N-degron pathway, whose first steps require the presence of O_2_ and NO for the correct functionality. C refers to unoxidized Cys and C* indicates the formation of Cys-sulfinic acid. Created with BioRender.com.

Plants are sessile organisms that have evolved to integrate external and internal signals in order to develop properly and to respond and adapt to different stresses. As plants are aerobic organisms, oxygen (O_2_) balance is required to maintain the correct control of respiratory energy supply. When there is a drop in O_2_ level, hypoxia is established, limiting aerobic respiration; anoxia occurs when O_2_ is completely absent in the environment (reviewed in Loreti and Perata 2020). Plants can experience hypoxia as part of their physiology, such as in meristematic tissues with high metabolic activity, or as an environmentally imposed stress, such as upon submergence and waterlogging (reviewed in Weits et al. 2021). Hypoxic stress signals are integrated via Group VII Ethylene Response Factors (ERF-VIIs), transcription factors that upregulate expression of core hypoxia response genes which direct acclimation to the hypoxic environment (Mustroph et al. 2009). In the case of submergence, this promotes survival, typically by entry into a quiescent state that reduces growth and respiration until O_2_ levels are restored when flooding recedes (reviewed in Voesenek and Bailey-Serres 2015; Hartman et al. 2019). The stability of the ERF-VIIs is regulated by the cysteine/arginine (Cys/Arg) branch of the N-degron pathway (Gibbs et al. 2011; Licausi et al. 2011; reviewed in Varshavsky 2024), which recognizes conserved methionine (Met) and Cys residues at the ERF-VII N-termini. Met cleavage followed by Cys oxidation allows N-terminal arginylation, ubiquitination, and degradation of the ERF-VIIs (or other targeted protein) (Fig. 1). Therefore, the enzymes that catalyze individual steps in Cys N-degron pathway (Fig. 1) are essential components of plant responses to these adverse environmental conditions. Interestingly, N-degron pathway mutants also indicate that ERF-VII stability is linked to other plant stresses in different species, such as drought, salinity, and oxidative stress (Vicente et al. 2017; reviewed in Dissmeyer 2019).

Protein N-termini provide an important site for approximately 65% of total protein processing, essential for regulation, localization, and, finally, degradation (reviewed in Bradshaw et al. 1998 and Varland et al. 2015). This process can occur co-translationally at the ribosome or post-translationally throughout the cell. Critical to the ability of the N-degron pathway to degrade ERF-VIIs and other Met-Cys-initiating target proteins is the generation of an oxidized Cys residue at the N-terminus. This relies on 2 families of enzymes: Met Aminopeptidases (MetAPs), which co-translationally catalyze the removal of the initial Met, and Plant Cys Oxidases (PCOs), which post-translationally catalyze the oxidation of the exposed Cys (Fig. 1). In this review we summarize what is known about the generation of N-degrons by MetAPs and PCOs, considering how their structures contribute to their interactions with substrates, how these are impacted by other cellular signals, as well as consideration of the role of this N-terminal processing through evolution in the plant kingdom.

**Advances**

- N-degron formation at Met-Cys–initiating protein termini could be an evolutionarily conserved system; phylogenetic and biochemical analyses indicate that MetAP and PCO enzymes are present throughout the plant kingdom.
- Further cellular or environmental signals may integrate at these N-degrons (e.g. additional oxidation of Nt-Cys).
- MetAP and PCO-catalyzed N-degron formation is not the only fate available to Met-Cys N-termini; they may be regulated by other post-translational modifications.
- ERFVII N-degron formation can be prevented with application of chemical inhibitors of PCOs.
- MetAP interaction with the nascent polypeptide–associated complex (NAC) could provide significant insights into co-translational enzymatic processing on eukaryotic ribosomes.

**Outstanding Questions**

- What additional environmental and physiological factors impact the activity of MetAPs and PCOs? How do these affect N-degron formation and protein stability?
- Genes encoding PCO-like enzymes are present throughout the plant kingdom, yet the established biological role for these enzymes involves substrates which are only present in vascular plants. Do PCO-like enzymes have an important role in nonvascular plants, and does it involve oxygen-sensing?
- What are the roles of different PCO and MetAP enzymes within the same organism? Are they sensitive to different cues or selective for different substrates?
- How frequently are Met-Cys N-termini “hidden” from MetAPs and/or PCOs through inter- or intra-protein interactions? Could the dynamics of such interactions control protein stability?
- Do N-termini–modifying enzymes form superchanneling complexes? Might these be dynamic in response to internal or external signals?
- Can MetAP/PCO activity be manipulated to control outcomes—for example, improve submergence resilience or regulate meristematic growth?

## Biological role of MetAPs and PCOs during plant physiology and stress

All proteins synthesized at the ribosome start with an initiating Met residue (or formyl-Met in prokaryotes). N-terminal Met/fMet excision (NME) is conserved from bacteria to eukaryotes, and the rules governing whether Met is removed or retained are similar in all organisms (reviewed in Meinnel et al. 1993; Walker and Bradshaw 1998). In eukaryotes, Met removal usually occurs when the nascent polypeptide is 45 to 82 residues long (optimal window for NME) (Sandikci et al. 2013; Yang et al. 2019) and when the second amino acid is conformationally favorable (usually small, see below). MetAPs catalyze cleavage of this initiating Met to leave the subsequent residue exposed. Depending on the fate of the Met-cleaved protein, this can have a diverse range of impacts in plant physiology.

Eukaryotes have 2 different groups of MetAPs, MetAP1 and MetAP2, and both types are known to be required for correct growth in plants, with the demand for cytoplasmic NME increasing during development (Giglione et al. 2000; reviewed in Giglione et al. 2004; Ross et al. 2005). As may be expected, *MetAP* transcripts are present during the whole plant life cycle and in all tissues. More specifically, *MetAP2B* transcripts are present in all tissues, while *MetAP1A* is induced in early developmental stages (except in the siliques), highlighting some specificities during plant growth (Ross et al. 2005).

MetAPs are located in all the cellular compartments where protein synthesis takes place (i.e. cytoplasm, plastids, and mitochondria) (Table 1, Ross et al. 2005). In *Arabidopsis thaliana*, as well as other species, there are 2 distinct types of cytosolic MetAPs, MetAP1A and 2 variants of MetAP2: MetAP2A and MetAP2B (Giglione et al. 2000; reviewed in Giglione et al. 2004). These appear to be functionally interchangeable in plants; no strong phenotypic differences have been observed when comparing wild-type plants with knockout mutants of *MetAP1A* or by specific inhibition of the MetAP2 group, but impairment of both at the same time drastically affects many stages of plant development (Ross et al. 2005). Fumagillin selectively inhibits the activity of cytosolic MetAP2s in eukaryotes, so the role of MetAPs can be determined through fumagillin treatment in combination with *MetAP1A* knockdown mutants. Using this approach, low concentrations of fumagillin applied to *A. thaliana metap1a* mutants decreased MetAP2 activity and were linked to strong alterations in the root system in *A. thaliana*. When MetAP2 activity is inhibited in *MetAP1A* knockdown mutants, developmental arrest, abnormal leaves, and reduction in hypocotyl elongation have been observed (Ross et al. 2005). During seed germination, NO responsiveness is also reduced when cytoplasmic MetAP activity is inhibited in *metap1a* mutants (Gibbs et al. 2014), triggering insensitivity during hypocotyl elongation and stomata NO-related responses (Gibbs et al. 2014). In contrast to MetAP1A and the MetAP2s, MetAP1B, MetAP1C, and MetAP1D are organelle specific (Giglione et al. 2003; reviewed in Giglione et al. 2004). Overall, therefore, MetAPs play a broad and fundamentally important role in regulating protein function in plant physiology.

**Table 1.** Subcellular localization of Arabidopsis MetAP and PCOs.

Name	TAIR entry	Subcellular localization	References
AtMetAP1A	At2g45240	Cytoplasm	Giglione et al. (2000)
AtMetAP1B	At3g25740	Chloroplast	Giglione et al. (2000)
AtMetAP1C	At1g13270	Chloroplast, mitochondrion	Giglione et al. (2000)
AtMetAP1D	At4g37040	Chloroplast, mitochondrion	Giglione et al. (2000)
AtMetAP2A	At2g44180	Cytoplasm	Giglione et al. (2000)
AtMetAP2B	At3g59990	Cytoplasm	Giglione et al. (2000)
AtPCO1	At5g15120	Cytoplasm, Nucleus	Weits et al. (2014)
AtPCO2	At5g39890	Cytoplasm, Nucleus	Weits et al. (2014)
AtPCO3	At1g18490	Mitochondrion	Predicted, https://suba.live/
AtPCO4	At2g42670	Nucleus	Predicted, https://suba.live/
AtPCO5	At3g58670	Nucleus	Predicted, https://suba.live/

TAIR, The Arabidopsis Information Resource.

MetAPs are known to catalyze removal of the N-terminal Met of MetCys-initiating proteins, meaning the exposed N-terminal Cys has the potential to become a substrate of the PCOs. These enzymes use O_2_ as a co-substrate to catalyze the oxidation of the N-terminal Cys of target proteins to N-terminal Cys-sulfinic acid, which in turn is a substrate for subsequent arginylation and the Cys N-degron pathway (Weits et al. 2014; White et al. 2017). In hypoxic conditions, PCO activity is reduced, thereby stabilizing N-terminal Cys substrates by impeding their progress along the Cys N-degron pathway. In *Arabidopsis*, the *PCOs* are expressed to a medium level in all tissue types in both cytoplasm and nucleus (Weits et al. 2014) (Table 1), with AtPCOs 1 and 2 predicted to contain nuclear localization signals (Weits et al. 2023). Expression of genes encoding AtPCOs 1 and 2, as well as PCOs from crops (Weits et al. 2014; Reynoso et al. 2019), is upregulated in response to hypoxia, suggestive of a feedback mechanism that allows rapid degradation of target proteins upon reoxygenation. This amplification in response to hypoxia is a trait observed in an evolutionarily conserved subclade of PCOs called B-type PCOs, which appear to arise in spermatophytes, whereas A-type PCOs are conserved throughout the plant kingdom (Weits et al. 2023). Notably, in Arabidopsis, both A- and B-type PCOs are required for complete regulation of the anaerobic response.

The PCOs have a much more limited substrate repertoire than the MetAPs, with no evidence that they catalyze oxidation of all MC-initiating proteins. However, it is well-documented that their oxygen-dependent activity regulates ERF-VII stability and that this enables plants to respond to hypoxic stress induced by submergence (Gibbs et al. 2011; Licausi et al. 2011; Weits et al. 2014; White et al. 2017). Only 2 other biologically validated substrates have been reported to date, Vernalization 2 (VRN2) (Gibbs et al. 2018), which is involved in the epigenetic regulation of vernalization, a key development step that promotes the transition of flowering plants to a reproductive state following prolonged exposure to cold temperatures, and Little Zipper 2 (ZPR2) (Weits et al. 2019), which controls transcription factors in the hypoxic niche of the shoot apical meristem that are integral to shoot apical meristem activity (Fig. 1). Kinetic studies of *A. thaliana*'s 5 PCOs place their O_2_ sensitivity (at least when reacting with ERF-VIIs) within physiologically relevant ranges for plant tissues, meaning they can be defined as O_2_ sensors (White et al. 2018). As with other eukaryotes, a plant's ability to sense O_2_ through PCO activity on selected N-terminal Cys proteins is therefore fundamental to their survival. This ability allows them to respond to low O_2_ levels arising as a result of abiotic and biotic stresses as well as during developmental stages (Gibbs et al. 2011; Licausi et al. 2011; Weits et al. 2014). Interestingly, a recent study suggests that oxygen-sensing by PCOs is also fine-tuned to local atmospheric O_2_ levels, thereby adapting plants for survival at different altitudes (Abbas et al. 2022).

## Structural features of MetAPs and PCOs

**Figure 2.**
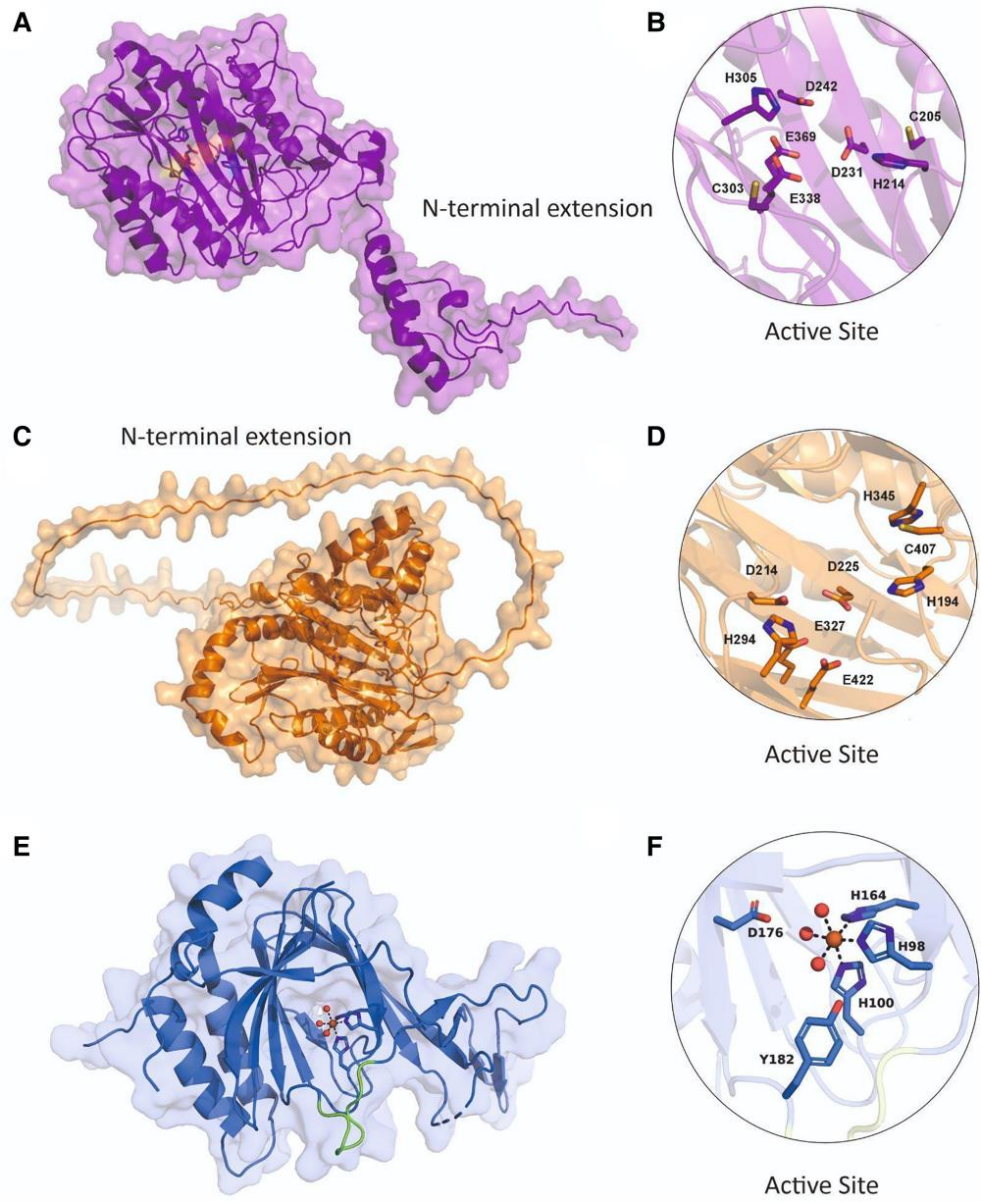
Predicted and reported structures of MetAPs and PCO4 from *Arabidopsis thaliana* showing overall folds and active site residues. **A, B)** AtMetAP1A (Alphafold2, UniProt ID: Q9SLN5). **C, D)** AtMetAP2A (Alphafold2, UniProt ID: Q9FV49). **E, F)** AtPCO4 (PDB: 6S7E; UniProt ID: Q9SJI9). Key active site residues are displayed as sticks. For AtPCO4, active site Fe (sphere) coordinated to 3×water molecules (spheres) and 3×His (sticks).

The MetAPs are monomeric proteins with a size ranging from approximately 27 to 60 kDa, varying based on their origin and class. They are metalloproteases, activated in vitro by various divalent cations like Fe^2+^, Co^2+^, Mn^2+^, and Zn^2+^, without altering the structure of their active site regardless of the specific metal ion bound (reviewed in Mucha et al. 2010). The nature of the metal cation present under physiological conditions remains uncertain for both MetAP1s (Walker and Bradshaw 1998; D’Souza and Holz 1999; D’Souza et al. 2002) and MetAP2s (Yang et al. 2001). MetAP1s and MetAP2s play identical enzymatic roles and have very similar 3D structures (Fig. 2, A to D), despite their amino acid sequences differing considerably. MetAPs of the same type (e.g. MetAP2A and MetAP2B) show high levels of sequence identity (reviewed in Lowther and Matthews 2002 and in Giglione et al. 2004). MetAP2A and MetAP2B have identical intron-exon organizations, produce mRNAs of the same size, their open reading frames share 86% sequence identity (Giglione et al. 2000), and their catalytic domains are more than 90% identical. In contrast, the main structural difference between MetAP1s and MetAP2s is an additional 60-residue α-helical region within the C-terminal portion of MetAP2s. Eukaryotic MetAPs differ from prokaryotic MetAPs by consistently featuring N-terminal extensions ranging from 50 to 100 residues (reviewed in Meinnel et al. 2006). These extensions can contain a preserved zinc motif in MetAP1s (Zuo et al. 1995; Giglione et al. 2000) or elongated sequences of basic and acidic residues in MetAP2s (Arfin et al. 1995; Li and Chang 1995) (Fig. 2, A and C).

MetAPs belong to the clan MG or M24 protease family (reviewed in Rawlings and Barrett 1995; Rawlings et al. 2002) and share a common structural feature known as the “pita-bread” fold, which is conserved in both classes and includes specific residues crucial for metal binding (Liu et al. 1998; Tahirov et al. 1998). It consists of 2 β-sheets surrounded by 4 α-helices and features a pseudo 2-fold axis of symmetry, which enables their narrow substrate specificity (Roderick and Matthews 1993). The interface between the N- and C-terminal sheets contains the active site involved in catalysis and metal ion binding, flanked by well-defined substrate binding pockets (Bazan et al. 1994). Despite the overall symmetry of the protein, the active site is asymmetrically located, with the Met binding pocket primarily formed by residues from the N-terminal sheet (Lowther et al. 1999). Crystallographic examinations of MetAPs have demonstrated that their substrate selectivity arises from the small size of the binding pocket, preventing the inclusion of amino acids with large side chains (Roderick and Matthews 1993; Liu et al. 1998).

The 3D structures of the enzymes within this enzyme family exhibit shared metal binding sites consisting of 5 residues (1 His, 2 Asp, and 2 Glu; Fig. 2, B and D) and a similar active site topology, featuring additional conserved His and Glu residues. This structural resemblance persists despite the limited amino acid sequence similarity among the diverse members of this enzyme family (reviewed in Bradshaw et al. 1998; Liu et al. 1998; reviewed in Lowther and Matthews 2000). In silico modeling of the active binding site of AtMetAP2 bound to fumagillin has suggested that all the amino acid residues essential for fumagillin recognition, including the critical His residue (His194 in AtMAetP2A, Fig. 2D) responsible for the covalent binding of this drug, are preserved in both plant MetAP2s (Ross et al. 2005). There is no evidence to date of post-translational modifications regulating the activity of MetAPs in plants. In human MetAPs, however, it has been reported that redox regulation of a key disulfide bridge in HsMetAP2A can impact its activity. The formation of a disulfide bridge between Cys228 and Cys448 results in inhibition, as this disulfide bond is 3 residues distant to a catalytic His231 residue (Chiu et al. 2014). Structural models of AtMetAP1A and AtMetAP2A suggest potentially important Cys and His residues close to the active site, which may be important, but no function has been ascribed to these residues to date. These include Cys205, His214, and Cys303 for MetAP1A (Fig. 2B) and His194, His345, and Cys407 for MetAP2A (Fig. 2D). NO can bind the Fe^2+^ from MetAP2 in *Pyrococcus furiosus*, although the effect on its activity is unknown (Copik et al. 2005).

Recent structural studies have revealed that these N-terminal extensions of eukaryotic MetAPs play crucial roles in ribosome binding and function. In a study performed by Gamerdinger et al. (2023), it was shown that MetAP1's N-terminal extension contains zinc-finger motifs that interact with the C-terminus of the nascent polypeptide–associated complex (NAC)-beta subunit, facilitating its association with the ribosome. This arrangement allows for co-translational processing of nascent chains when they reach approximately 50 amino acids in length. By using multiple model organisms (yeast, *Caenorhabditis elegans*, and human cells), Gamerdinger et al. (2023) demonstrated the conservation of this mechanism throughout eukaryotic evolution. Similarly, studies in human and fungal systems showed MetAP2’s N-terminal extension, with its poly-charged regions, interacts with the ribosomal RNA segment ES27L, positioning it near the exit tunnel for efficient protein processing. Notably, autoproteolytic cleavage of this extension significantly alters MetAP2's ribosome binding dynamics and function (Klein et al. 2024a). Recent studies have provided significant insights into co-translational enzymatic processing on eukaryotic ribosomes. In mammalian cells, NAC assembles a multienzyme complex with MetAP1 and N-acetyltransferase A early during translation, pre-positioning their active sites for timely processing of nascent proteins and regulating their activity (Lentzsch et al. 2024). Complementarily, Klein et al. (2024b) used cryo-EM to identify dynamic assemblies of ribosome-associated factors on human 80S ribosomes, including MetAPs and N-terminal acetyltransferases, which enhance NME and N-terminal acetylation efficiency (Klein et al. 2024b). In human cells, the MetAP-like protein Ebp1 occupies a position at the ribosomal tunnel exit similar to that of MetAPs, interacting with various ribosomal components (Wild et al. 2020). These findings confirm earlier in vitro assays, suggesting the importance of these N-terminal extensions for activity and ribosome interactions (Vetro and Chang 2002), while providing detailed structural insights into their specific roles in protein-ribosome and protein-RNA interactions.

Crystal structures have been resolved for 3 of the 5 PCO isoforms present in Arabidopsis, AtPCO 2, 4, and 5 (White et al. 2020; Chen et al. 2021), which revealed them also to be monomeric enzymes (Fig. 2E). The structures reveal a double-stranded β-helix, which identifies them as part of the cupin superfamily of proteins. The double-stranded β-helix is flanked on one side by N-terminal α-helices and on the other by a C-terminal loop region. The β-barrel core creates a channel through the center of PCO, within which lies the active site. A nonheme Fe(II) is an essential active site component and is held in position by 3 coordinating His residues. In the absence of a substrate, its octahedral geometry is completed by 3×H_2_O molecules (Fig. 2F). This core structure is conserved in 2-aminoethanethiol dioxygenase (ADO), a human paralogue of the PCOs, as well as in small molecules thiol dioxygenases (reviewed in Gunawardana et al. 2022).

No substrate-bound structures of the PCOs or ADO have been resolved to date, and so there is limited knowledge on how Cys-initiating substrates interact with Fe and surrounding active site residues. However, spectroscopic studies of ADO in complex with its small molecule substrate cysteamine indicate monodentate binding to the Fe via the Cys thiol (Fernandez et al. 2020; Wang et al. 2020). Oxygen can then bind to the Fe, where it becomes activated and reacts with the thiol sulphur to form Cys-sulfinic acid (reviewed in Gunawardana et al. 2022). Given the high sequence and structural similarity between PCO and ADO, monodentate binding might also be expected for PCO. This is supported by docking experiments conducted for AtPCO4 (PDB: 6S7E) and RELATED TO AP (RAP)2.12_2–15_ (Lavilla-Puerta et al. 2023). In addition, the docked structure displays a hydrogen bonding interaction between the amino group of the N-terminal Cys of the substrate and the Asp176 in the active site (AtPCO4 numbering); this may help stabilize and orient the substrate in the active site. Asp176 is conserved among PCOs and ADO, and site-directed mutagenesis experiments highlight its importance as variants Asp176Asn in AtPCO4 and Asp176Ala in AtPCO5 show a significant drop in AtPCO4 activity (White et al. 2020; Chen et al. 2021). Reduced AtPCO4 activity has also been observed following mutagenesis of Tyr182 to Phe, albeit less pronounced than for Asp176Asn (White et al. 2020). The AtPCO4 (6s7e) and RAP2.12_2–15_ docked structure displays hydrogen bonding between the Tyr182's hydroxyl group and the substrate backbone, pointing toward a substrate binding role for Tyr182.

Crystal structures of the PCOs and ADO display 2 access routes to the active site in the center of the β-barrel channel. A narrow route is speculated to play a role in O_2_ delivery to the Fe(II), whereas docked structures indicate that wider, more open entry may accommodate binding of N-terminal Cys protein substrates (Fernandez et al. 2022). In small molecule thiol dioxygenases such as cysteine dioxygenase (Fernandez et al. 2022), the substrate entry channel is partially obstructed by a hairpin loop region. In both PCO and ADO (White et al. 2020; Fernandez et al. 2021; Wang et al. 2021), the equivalent loop (residues 182-190 and 212-220, respectively) appears to be positioned to promote access of larger substrates to the active site (reviewed in Gunawardana et al. 2022). This loop region may be of structural significance; it is located near the proposed substrate binding channel; sequence divergence/variation between PCOs in this region and the substrate trajectory in the docked structure are suggestive of a role in substrate recognition by this loop region.

There is no evidence to date for PCO involvement in complex formation. Nevertheless, it has been proposed that “superchanneling complexes” may exist for Arg/N-degron complexes in humans and yeast to ensure efficient processing (Oh et al. 2020). Whether such complexes form in plants between PCOs and other N-degron machinery remains to be seen; rapid processing would ensure prompt degradation of target proteins, but involvement in a complex could also restrict O_2_ access to PCO active sites, reducing their efficiency.

## Structural requirements for MetAP and PCO substrate recognition

As all proteins are biosynthesized with initiating Met residues, but not all are cleaved, MetAPs must show preference for certain proteins. It is well established that MetAPs recognize not only the N-terminal Met residue but also the surrounding structural features of the substrate protein. The presence of secondary structures (α-helices or β-strands) as well as specific sequence motifs near the N-terminal region determines how accessible the Met is to MetAPs. The main factor that determines if the Met will be removed or not is the nature of the second amino acid. Met will be retained if the second residue is bulky or has an electrically charged side chain. In contrast, complete cleavage of the Met will occur when the nonbulky, uncharged residues Ala, Cys, Gly, Pro, or Ser occupy the second position (reviewed in Sherman et al. 1985). Meanwhile, Thr or Val in that position have been associated and further proven to correlate with a less predictable Met cleavage (Frottin et al. 2006, 2016).

Winter et al. (2021) reviewed the amino acid frequency and distribution of the first 30 residues in *A. thaliana* proteins. Within the subset of proteins predicted to localize in the cytoplasm and nucleus (where enzymes of the N-degron pathway act) there are 68, 378, and 155 proteins starting with Met-Cys, Met-Asn, and Met-Gln, respectively. In the case of these proteins, the Met can be removed by MetAP1A or MetAP2 group enzymes, exposing a tertiary destabilizing residue that could be potentially modified by PCOs in the case of the Cys (Weits et al. 2014; White et al. 2017) or deamidase enzymes for Asn and Gln (Vicente et al. 2019). These numbers highlight the potential Cys N-degron pathway substrates in plants that remain to be discovered. The software TermiNator3 predicts N-terminal Met excision as well as N-terminal acetylation, myristoylation, and S-palmitoylation of either prokaryotic or eukaryotic proteins and is a helpful tool that may contribute to identification of plant MetAP substrates (Frottin et al. 2006; Martinez et al. 2008).

Just as MetAPs require their substrates to be sufficiently unstructured that the N-terminal residues can bind in the active site, PCOs also require unstructured N-termini to allow access to the active site through a relatively narrow channel (White et al. 2020). Of the 246 gene models in the Arabidopsis genome that initiate Met-Cys and the 68 predicted to localize in the nucleus or cytoplasm, only 7 have been confirmed to date as bona fide PCO substrates (described above and reviewed in Holdsworth et al. 2020). While these numbers may be indicative of yet to be discovered PCO substrates, they may also hint at mechanisms of protection from PCO-mediated oxidation. Prevention of Met removal by MetAPs (e.g. by inhibition) or post-NME N-terminal modifications could prevent oxidation; N-terminal acetylation has been linked to protein stabilization in plants (Linster et al. 2022) and animals (Mueller et al. 2021), and Nt-acetylation has recently been shown capable of shielding Cys-initiating substrates from ADO-catalyzed Nt-Cys oxidation in vitro (Heathcote et al. 2024). Steric evasion could also prevent PCO-catalyzed oxidation through “hidden” N-terminal Cys; SUB1A-1, a rice ERF-VII, likely escapes Cys N-degron pathway–mediated degradation through physical interaction between its N- and C-termini, preventing adequate exposure of the N-terminus (Lin et al. 2019). Hidden N-termini for other substrates could arise through such intra- or indeed inter-molecular complexes.

While PCOs have thus far only been shown to recognize and interact with the N-terminal Cys of their substrates, molecular docking is suggestive of recognition and/or binding of the aforementioned hairpin loop to amino acids following the N-terminal residue. In vitro kinetic studies with peptidic substrates suggest that the 5 AtPCOs show substrate preferences within the ERF-VII substrates. Given the conservation of the N-termini of the ERF-VII family, any substrate preferences are likely to arise from subtle variations in the peptide sequences beyond the conserved N-terminal region and their interaction with the hairpin loop and other regions of the PCOs (White et al. 2020). In biochemical experiments, AtPCO4 showed a striking preference for oxidation of AtRAP2_2-15_ over ZPR2_2-15_ and VRN2_2-15_ (Taylor-Kearney et al. 2022b), which do not share sequence conservation with the ERF-VII, instead having notably different sequences following the N-terminal Cys. It will be interesting to know whether this preference is retained in the other AtPCOs, which would be indicative of ERF-VII homeostasis being, at least kinetically, the primary function of the AtPCOs.

## Evolutionary perspective of MetAPs and PCOs

The excision of initial Met amino acid by the MetAPs has been observed in all living kingdoms, showing an important evolutionary conservation of this process (reviewed in Giglione et al. 2004). MetAP activity has been reported in several organisms with different developmental complexity, from the simplest such as *Escherichia coli* or *Salmonella typhimurium* (Chang et al. 1989; Miller et al. 1989)—in which the mutation of this gene is lethal—as well as in yeast (Li and Chang 1995), to organisms on a higher evolutionary scale, including monocot and dicot plants as well as humans (reviewed in Datta 2000; Bernier et al. 2005; Morgante et al. 2009). These examples show phylogenetic conservation and the relevance of the maintenance of these proteins during evolution.

**Figure 3.**
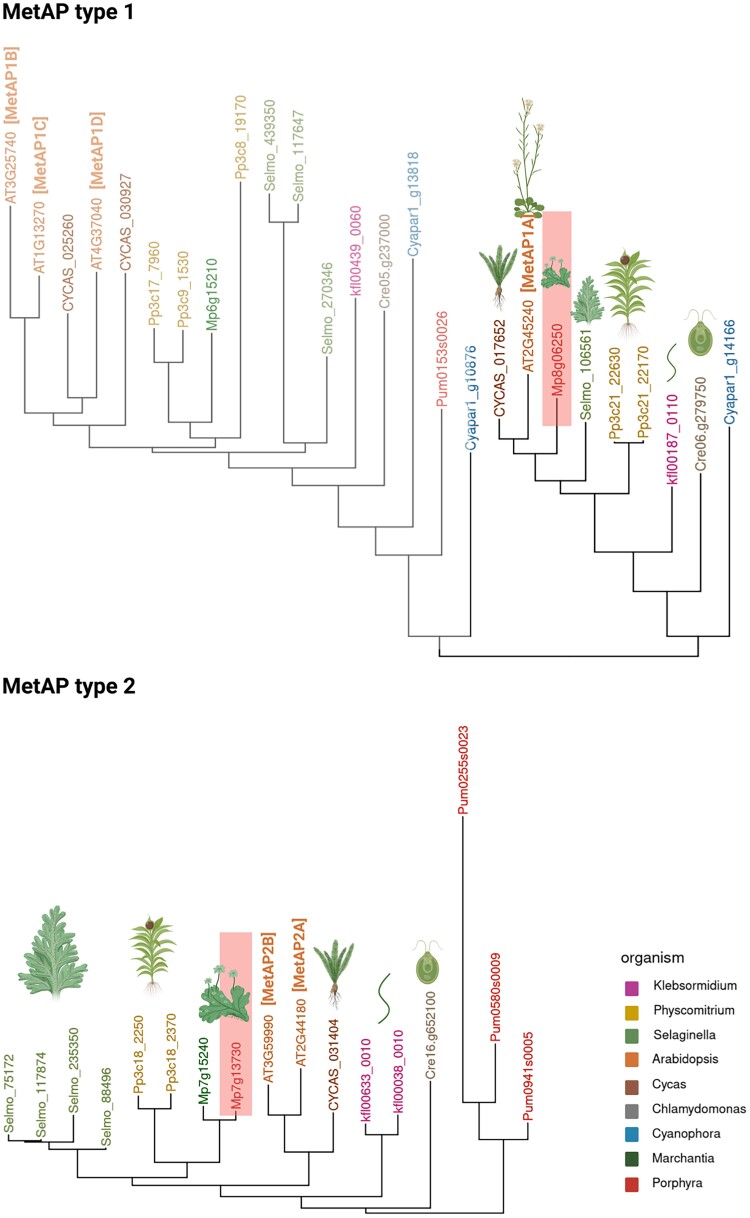
Phylogenetic tree of MetAPs in the land plants lineage. Phylogenetic tree performed by AlgaeFUN (Romero-Losada et al. 2022) with MetAPs type 1 (left) and MetAPs type 2 (right). The phylogenetic tree is based on Mp8g06250 (left) and Mp7g13730 (right) Marchantia protein sequences (highlighted). Color legend indicates the organisms selected from bryophytes to angiosperms, also represented by pictures. Created with BioRender.com.

Interestingly, MetAP subclasses MetAP1 and MetAP2 are not equally represented in prokaryote and eukaryote organisms, showing a ubiquitous phylogenetic distribution. In this regard, prokaryotes present only 1 MetAP isoform, MetAP1, but eukaryotes express both MetAP1 and MetAP2 (Bernier et al. 2005). In plants, partial sequences encoding homologs of *MetAP1A* and *MetAP2* have been identified in various crops, including rice, alfalfa, soybean, tomato, sorghum, corn, and pine. This suggests that the MetAP sequence set obtained from the model plant *A. thaliana* represents the general situation in other flowering plants, both dicots and monocots (Giglione et al. 2000) (Fig. 3). During plant evolution, the study of bryophytes corresponding to basal land plant lineages helps us to understand the conservation of the molecular mechanisms during terrestrial plant evolution. Analysis of the liverwort *Marchantia polymorpha* genome shows the presence of 1 *MetAP1* ortholog (*Mp8g06250*) and 2 *MetAP2* orthologs (*Mp7g13730* and *Mp7g15240*) (Bowman et al. 2017). A phylogenetic tree generated using AlgaeFUN (Romero-Losada et al. 2022) to compare these MetAP1 and MetAP2 Marchantia orthologs with representative species of other bryophytes (*Physcomitrella patens*), lycophytes such as *Selaginella moellendorffii*, the gymnosperm Cycas, and the angiosperm *A. thaliana* (Fig. 3) shows a clear separation in the MetAP type 1 of proteins that are located in different cellular localization in *A. thaliana* (Table 1). On the other hand, the MetAP type 2 phylogenetic tree shows only 1 ortholog to Cycas, while the rest of species contain at least 2 proteins in this group (Fig. 3).

MetAP1 sequences in humans and plants appear to be closer to the MetAP1 sequence from *E. coli* than to the MetAP2 sequences of the species itself, most notably when looking at the substrate pocket (reviewed in Heinrich et al. 2023). The substrate specificity of the MetAP1s, determined by the first amino acid residue of the target proteins and the sequence between the second and fifth amino acids, is also similar in both bacteria and humans (Xiao et al. 2010). Related to specificity, the most conserved residue in MetAP1 is a Cys residue located in the protein active site, which is important in binding Met residues. However, a new subtype of MetAP type 1 that has asparagine in the active site instead of Cys has been identified in proteobacteria (Bala et al. 2019). Microbes with MetAPs containing this asparagine residue are placed in a phylogenetically unique position. In plants, when comparing the sequence of the MetAP1a protein of *E. coli* with the sequence of the MetAP1 proteins of plant species analyzed in Fig. 3, it is observed that Cys 70, which is involved in specificity, is also conserved.

**Figure 4.**
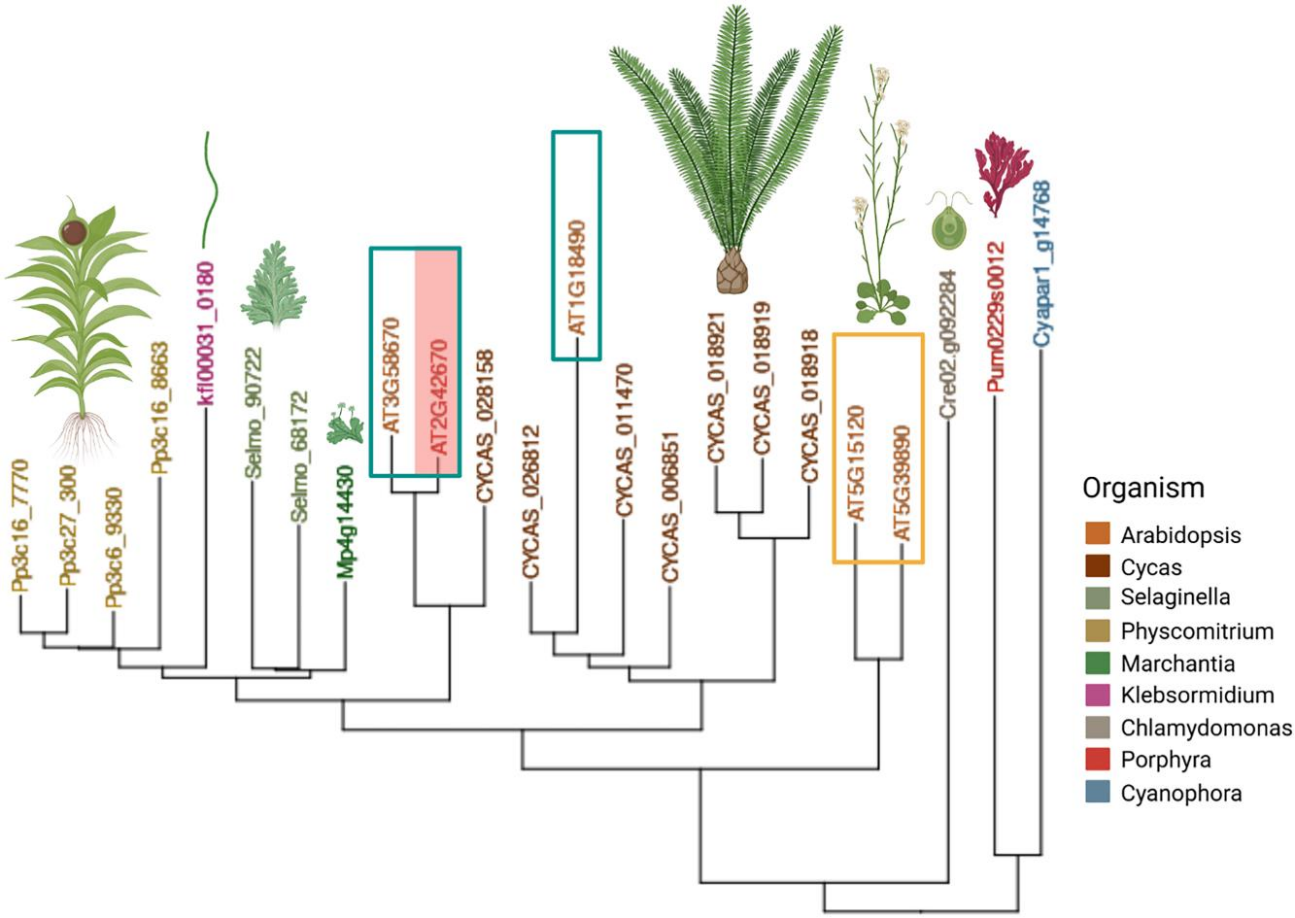
Evolutionary conservation of PCOs in the Archaeplastida kingdom. Phylogenetic tree performed by AlgaeFUN (Romero-Losada et al. 2022), based on AtPCO4 protein sequence (AT2G42670, highlighted). Color legend indicates the organisms selected, also represented by pictures. Boxes mark A-type (3, 4, 5) and B-type (1, 2) AtPCOs respectively. Created with BioRender.com.

While MetAPs are found in all kingdoms, sequences consistent with N-terminal cysteine dioxygenases (NCOs) have only been confirmed in eukaryotes. NCOs are present in both plant (PCO) and animal lineages (ADO), and structural and functional studies show enzymes from these lineages are analogous. Phylogenetic studies show that while PCO and ADO sequences are broadly conserved across Archaeplastida and Animalia, they are almost entirely absent from all fungus phyla, except for Chytridiomycota (Weits et al. 2023). While the presence of NCOs in chytrid species could be interpreted as the loss of enzymatic function in other fungal phyla, phylogenetic analysis of these sequences shows a closer association with plant PCOs than to metazoan ADOs, suggesting an origin via horizontal transfer of a PCO-like gene from an ancestral green organism to a chytrid progenitor (Weits et al. 2023). Identification of PCO-like sequences in Glaucophytes, Rhodophytes, and Viridiplantae displays the ancient origin of PCOs (Fig. 4), potentially predating the Last Plastid Common Ancestor of the Archaeplastida. As described above, B-type PCOs arose in spermatophytes, whereas A-type is more ancient. The increasing number of PCO-coding genes in flowering plants correlates with increased genome size in plants; while ratios of B-type to A-type PCO vary considerably between species, each clade is present within all spermatophytes (Weits et al. 2023). Interestingly, the role of PCOs in organisms arising earlier than vascular plants is not clear due to the absence of confirmed substrates beyond tracheophytes (ERF-VIIs) and Angiospermae (VRN2 and ZPR2) (Weits et al. 2023). While phylogenetic and structural analysis displays the potential ancient origin of functional PCOs, Nt-Cys dioxygenase activity has only been confirmed in vitro up to Bryophyta and Charophyta (Taylor-Kearney et al. 2022b), limiting proven enzyme activity to date to organisms that emerged with Streptophyta. Characterization of PCO function in more basal groups, along with identification of native substrates, is still required to provide a greater understanding of the origin of PCO enzymes and their function.

## Future perspectives

Overall, the combined activity of plant MetAPs and PCOs initiates the controlled destabilization of specific proteins in response to environmental cues. These activities are important for ERF-VIIs, VRN2 and ZPR2 regulation in response to acute hypoxia, as experienced upon submergence, or chronic hypoxia, but also in aspects of plant development. There also may be other, as yet unidentified Met-Cys–initiating proteins that are similarly regulated by these proteins, particularly in basal plant species. As well as being responsive to O_2_, which has been well-characterized, the possibility also exists that other modulators of these enzymes can also impact the stability of their targets. One notable example is NO, which has been shown to be necessary for degradation of targets of the Cys/Arg-branch of the N-degron pathway through an as yet unidentified mechanism (Gibbs et al. 2014). Additional post-translational modifications of Cys N-degron pathway enzymes or targets may play an important role in efficient destabilization; indeed, a recent report indicates that ERF-VII N-termini are oxidized by both PCOs and an additional component that results in formation of Cys-sulfonic acid, before or after arginylation (Zubrycka et al. 2023).

Climate change is increasing the frequency and intensity of extreme weather, including flooding events. ERF-VII stabilization is important in temporary adaptation to hypoxic conditions to promote survival. Modulation of MetAP or PCO activity towards these substrates therefore has the potential to improve plant flood tolerance. The PCOs are particularly good targets for this given there is less potential for off-target effects. Complete inhibition of PCOs via permanent ERF-VII stabilization (Licausi et al. 2011) or in *PCO* knockouts has been shown to have detrimental effects on plant survival (Masson et al. 2019). However, there is evidence that an approach that tailors PCOs O_2_ sensitivity or substrate selectivity either via genetic modification or chemical treatment could promote plant tolerance to hypoxic conditions (reviewed in Taylor-Kearney and Flashman 2022a). Indeed, the first chemical inhibitors of AtPCO4 have recently been identified that show an improved recovery post anoxia in treated seedlings (Lavilla-Puerta et al. 2023). Deeper understanding of MetAP and PCO biochemical and biological functions will support these endeavors.

## Data Availability

Data available from corresponding authors upon request.

## Dive Curated Terms

The following phenotypic, genotypic, and functional terms are of significance to the work described in this paper:

VRN2 Gramene: AT4G16845
VRN2 Araport: AT4G16845

biological role CHEBI: CHEBI:24432

fumagillin CHEBI: CHEBI:48635

oxygen CHEBI: CHEBI:25805

ZPR2 Gramene: AT3G60890
ZPR2 Araport: AT3G60890

PCO1 Gramene: At5g15120
PCO1 Araport: At5g15120

RAP2.12 Gramene: AT1G53910
RAP2.12 Araport: AT1G53910

AtMetAP1A Gramene: At2g45240
AtMetAP1A Araport: At2g45240

AtMetAP2A Gramene: At2g44180
AtMetAP2A Araport: At2g44180
